# Associations of HEXACO and dark traits with power and gender harassment

**DOI:** 10.3389/fpsyg.2026.1816134

**Published:** 2026-06-17

**Authors:** Eisuke Tokiwa

**Affiliations:** SUNBLAZE Co., Ltd., Tokyo, Japan

**Keywords:** dark triad, harassment, HEXACO model, non-WEIRD, workplace bullying

## Abstract

Workplace harassment carries substantial individual and organizational costs, yet the roles of antagonistic ‘dark’ traits and broader personality remain debated. In a cross-sectional online survey of 354 currently employed Japanese adults recruited via Lancers, we tested whether HEXACO traits—especially Honesty–Humility (H–H)—are associated with self-reported power and gender harassment perpetration tendencies beyond the Dark Triad (Machiavellianism, narcissism, psychopathy), and whether predictor structure differs by sex. Participants completed the HEXACO-60, the SD3-J, and validated harassment measures. HC3-robust hierarchical regressions showed that psychopathy was positively associated with power-harassment perpetration tendencies (*β* ≈ 0.32–0.40, *p* < 0.001), whereas H–H was negatively associated (β = −0.14, *p* = 0.049). For gender-harassment perpetration tendencies, narcissism and psychopathy were positive predictors, while H–H and Openness were negative; Machiavellianism showed a negative coefficient in the multivariate models. Adding HEXACO traits explained additional variance beyond Dark Triad-only models (ΔR^2^ = 0.032, *p* = 0.036 for power; ΔR^2^ = 0.096, *p* < 0.001 for gender). Sex-stratified analyses revealed a meaningful divergence in predictor structure: among men, Openness was the dominant negative predictor of gender-harassment perpetration tendencies (*β* = −0.44, *p* < 0.001), whereas among women H–H and Machiavellianism (negative, conditional on the other Dark Triad traits) were the most prominent predictors. We interpret these findings cautiously: incremental variance from H–H is consistent with—but does not by itself establish—a moral-personality contribution distinct from antagonistic traits, an inference that ultimately requires latent-variable modeling. Findings are framed as patterns of association in a Japanese employee sample rather than as evidence of universal mechanisms.

## Introduction

### The dark triad

Narcissism, Machiavellianism, and Psychopathy—the Dark Triad (Paulhus and Williams, 2002)—share a core of selfishness and reduced empathy while differing in motivational and behavioral profile. Narcissism centers on grandiosity, admiration-seeking, and hypersensitivity to ego threat (Bushman and Baumeister, 1998; Morf and Rhodewalt, 2001; Raskin and Terry, 1988). Machiavellianism reflects strategic manipulation, moral flexibility, and an instrumental interpersonal orientation (Christie and Geis, 1970; Jones and Paulhus, 2009). Psychopathy reflects callousness, weak guilt, impulsivity, and antisocial tendencies (Cleckley, 1941; Hare, 2003; Lynam and Derefinko, 2006). Across measures and contexts these three traits show robust convergent variance with one another (Vize et al., 2018) and stand in strong contrast to the Honesty–Humility (H–H) factor of the HEXACO model (Ashton and Lee, 2007; Lee and Ashton, 2005).

### The dark triad and harassment perpetration tendencies

The Dark Triad has been linked through distinct pathways to deviant workplace behavior, abusive supervision, counterproductive work behavior (CWB), and bullying perpetration (LeBreton et al., 2018; Mathieu et al., 2013; O'Boyle et al., 2012; Pletzer et al., 2019b). Psychopathy in particular has been associated with abusive supervision and “petty tyrant” patterns (Ashforth, 1994; Babiak and Hare, 2006; Boddy, 2011), with environmental moderators including leader sleep quality and emotional-intelligence deficits (Barnes et al., 2015). Machiavellianism has been associated with reduced organizational citizenship behavior and bullying perpetration (Baughman et al., 2012; Dåderman and Ragnestål-Impola, 2019), and with proclivity for sexual harassment under specific gender-role-relevant cognitions (Jonason et al., 2012; Zeigler-Hill et al., 2016). Narcissism has been associated with ego-threat aggression, sexual coercion, and CWB (Bushman et al., 2003; Grijalva, 2015; O'Boyle et al., 2012). These pathways converge on harmful workplace outcomes while differing in proximal mechanism, motivating analyses that simultaneously adjust for the three components rather than relying on bivariate associations alone. Importantly, because psychopathy is in part conceptually defined by antisocial tendencies, an unadjusted bivariate association between psychopathy and harassment perpetration is largely tautological; informative tests therefore concern (i) whether psychopathy retains a positive association after adjustment for the other two Dark Triad components and (ii) whether the pattern across outcomes is differential rather than uniform.

### HEXACO in personality research

The HEXACO model (Ashton and Lee, 2007; Ashton et al., 2004) extends the Five-Factor structure by adding Honesty–Humility (H–H), reorganizing Agreeableness and Emotionality, and providing a more orthogonal trait space (Thielmann et al., 2021). H–H captures sincerity, fairness, greed avoidance, and modesty—anti-egoistic and anti-manipulative variance that the Big Five only weakly captures (Howard and Van Zandt, 2020). H–H is consistently and strongly negatively related to the Dark Triad (Lee et al., 2013; Moshagen et al., 2018) and has shown incremental predictive validity for socially aversive workplace outcomes beyond the Big Five (Pletzer et al., 2019a). Emotionality reorganizes anxiety/dependence content while moving anger out of the domain (Ashton and Lee, 2009); meta-analytic and cross-cultural work shows women score higher than men on Emotionality, with smaller differences on the other HEXACO domains (Costa et al., 2001; Schmitt et al., 2008; Morales, 2020). Agreeableness in HEXACO emphasizes interpersonal regulation under provocation (low aggression, low revenge motivation; Ashton et al., 2014). Conscientiousness captures self-regulation and norm-compliance and is a robust correlate of citizenship behaviors and reduced CWB (Pletzer et al., 2021). Openness reflects intellectual curiosity, aesthetic sensitivity, and broader value orientation (Ashton and Lee, 2007). For the present study, H–H occupies the focal theoretical position as an inverse to the Dark Triad, while Agreeableness, Emotionality, and Openness provide additional, theoretically motivated adjusted predictors of harassment perpetration tendencies.

### HEXACO and workplace behaviors

HEXACO-based research has accumulated substantial evidence for the model in workplace contexts. H–H and Conscientiousness consistently predict reduced workplace deviance, abusive behavior, and CWB and increased citizenship behavior, particularly when assessed at the facet level or via situational-judgment instruments (Breevaart and de Vries, 2017; Lee et al., 2005; Pletzer et al., 2019b; Pletzer et al., 2021). Agreeableness suppresses CWB and is positively related to democratic, servant, and authentic leadership behaviors (Pletzer et al., 2019b; Zettler et al., 2020). Extraversion and Openness contribute to engagement, charismatic-leadership preferences, and adaptive learning (Anglim et al., 2019; Breevaart and de Vries, 2021). Within this framework, Honesty–Humility has been argued to be the single most informative HEXACO factor for socially aversive workplace outcomes (Lee et al., 2019), motivating its central position in the present analyses.

### Dark triad research in Japan

Although Dark-Triad research in Japan has expanded—including Japanese adaptations of short-form measures (Shimotsukasa and Oshio, 2017; Tamura et al., 2015) and studies on manipulative strategies, interpersonal aversion, gambling tendencies, loneliness, and prosociality (Koshinaka, 2020; Masui and Ura, 2018; Oda and Matsumoto-Oda, 2022; Takada, 2023; Tomii and Oshio, 2024)—systematic examinations of direct associations between Dark-Triad and HEXACO traits and workplace harassment perpetration in Japan remain scarce. This is consequential for cross-cultural generalization: effect sizes and pathways established in WEIRD samples (Henrich et al., 2010) may not directly transfer to Japanese organizational contexts. The present study contributes by directly examining HEXACO and Dark-Triad associations with self-reported power- and gender-harassment perpetration tendencies in a Japanese employee sample, while explicitly framing inference as patterns of association in this sample rather than as universal mechanisms.

### Purpose of the study

The present study examines how HEXACO traits and Dark Triad traits are jointly associated with self-reported workplace harassment perpetration tendencies among employed Japanese adults, and whether the associative structure differs across sex. Two distinct outcomes are considered: power-harassment perpetration tendencies (a multifaceted index combining behavioral enactment, attitudinal endorsement, and supervisor-centered interpersonal dynamics; Tou et al., 2017) and gender-harassment perpetration tendencies (commission and omission behaviors; Kobayashi and Tanaka, 2010). Two key analytic questions structure the inquiry: (i) Are the magnitudes and directions of personality–harassment associations observed in WEIRD samples reproduced in a Japanese employee sample? (ii) Does the HEXACO model—particularly Honesty–Humility (H–H)—account for additional variance over and above the Dark Triad and demographic covariates? Below we state our hypotheses in a form intended to be falsifiable rather than tautological. Reviewers have rightly noted that ‘psychopathy is antisocial; therefore psychopathy predicts harassment’ is not a substantively informative claim because the criterion overlaps with the predictor’s nominal definition. We therefore distinguish (a) the bivariate-level prediction (which would be unsurprising if observed and uninformative on its own), from (b) incremental and pattern-level predictions that would be informative if confirmed and would constrain the model if disconfirmed. Pattern-level hypotheses (the falsification targets): H1 (Differential Dark-Triad pattern). Psychopathy will retain a positive coefficient for power-harassment perpetration tendencies in adjusted models that simultaneously include Machiavellianism and narcissism. If, in adjusted models, psychopathy fails to retain a significant positive coefficient while one of the other dark traits dominates, H1 would be disconfirmed. H2 (Differential outcome). Psychopathy will be a stronger adjusted predictor of power-harassment perpetration tendencies than of gender-harassment perpetration tendencies, whereas narcissism will be more comparably represented across both outcomes. If psychopathy shows comparable or stronger adjusted association with gender-harassment than with power-harassment, H2 would be disconfirmed. H3 (Incremental HEXACO). Adding the six HEXACO factors over and above demographic covariates and the Dark Triad will yield a non-trivial increase in explained variance (informally, ΔR^2^ ≥ approximately 0.02–0.03 with a non-trivial F-change). If ΔR^2^ is approximately zero (or numerically positive but with a non-significant F-change at *α* = 0.05) for both outcomes, H3 would be disconfirmed. H4 (Direction within HEXACO). Among the HEXACO factors, H–H and Agreeableness will be negatively associated with both harassment outcomes in adjusted models. If H–H or Agreeableness is positively associated, or null, with both outcomes after adjustment, H4 would be disconfirmed. H5 (Sex-conditional structure). The relative ordering and significance of trait predictors will differ between men and women, particularly for gender-harassment perpetration tendencies, in line with documented mean-level sex differences in HEXACO Emotionality (Costa et al., 2001; Schmitt et al., 2008) and the differential context of gender-role enactment in Japanese workplaces. Comparable predictor structure across sexes for gender-harassment perpetration tendencies would disconfirm H5. We additionally test (exploratorily, not confirmatorily) Dark Triad × H–H interactions. We do not preregister directional predictions for these interaction terms; we report them descriptively. Two interpretive caveats are flagged here at the outset. First, the variance in H–H that remains after adjusting for the Dark Triad is, in this analytic framework, a residualized observed-score component; we describe it as an adjusted association, and we do not interpret it as a separable latent fairness/sincerity factor distinct from Dark-Triad antagonism, because such a specificity claim would require a latent-variable framework (e.g., bifactor or hierarchical CFA) that the present study does not estimate. Second, when one Dark Triad component (most likely Machiavellianism, given its strong covariance with the other two) shows a negative coefficient after adjustment, we interpret this as the well-documented behavior of overlapping predictors under multiple regression rather than as a substantive ‘protective’ role of that trait. Both caveats are revisited in the Discussion.

## Methods

### Research design and implementation

This study was a cross-sectional online survey. Data were collected via a Google Forms questionnaire in which the researcher configured every item as required (i.e., respondents could not submit the form without entering a response to every item; this is a per-form design choice in Google Forms, not an inherent property of the platform). The primary analyses were Spearman rank correlations and hierarchical multiple regression with HC3 heteroscedasticity-consistent standard errors.

### Participants

Participants were recruited through the Lancers crowdsourcing platform,^1^ and 380 individuals responded to the survey. Participation was restricted to currently employed adults; respondents were required at survey entry to confirm that they were presently employed, and those who indicated otherwise were not allowed to proceed. After excluding inattentive responses (e.g., the same option selected consecutively across long item blocks, extremely short or long total response times) and duplicate submissions according to predefined rules, the final analytic sample consisted of 354 currently employed Japanese adults (134 men, 220 women). The age distribution was 32 participants aged 18–19, 100 in their 20s, 124 in their 30s, 70 in their 40s, and 28 aged 50 or older. Although the sample included younger respondents, all participants confirmed current employment status, ensuring that responses to the harassment measures were grounded in actual occupational experience rather than hypothetical judgments. No homemakers or unemployed individuals were retained. Because every Google Forms item was configured as required by the researcher, item-level missingness from skipped items did not arise; a very small number of technical missing values (≤ 0.3% in any indicator) corresponded to data-cleaning anomalies (e.g., out-of-range entries flagged during validation) and were handled by complete-case analysis with negligible impact on the results. Managerial status (i.e., whether the respondent currently held a formally supervisory role with subordinates) was not assessed at intake and is therefore not available as a covariate; the implications of this for the interpretation of the power-harassment items are addressed in the Limitations.

### Ethical considerations

Prior to questionnaire administration, participants were provided with written information about the purpose of the study, the procedures, the voluntary nature of participation and their right to withdraw at any time without penalty, the privacy-protection procedures (including the separate storage and subsequent permanent deletion of crowdsourcing-platform user identifiers, the retention of only de-identified questionnaire data, and the public archiving of the de-identified dataset), and the planned dissemination of results. All participants provided written informed consent electronically through the Google Forms consent page presented prior to the questionnaire, before responding to any survey items. Crowdsourcing-platform user identifiers were temporarily linked to questionnaire responses solely for the purpose of disbursing participant compensation and were permanently deleted after payment, after which the dataset retained for analysis and public release contained no direct or indirect identifiers (the dataset was de-identified, not anonymously collected). The study protocol was reviewed and approved by the Institutional Review Board of the Public Health Research Foundation (reference no. 25G0001), an independent ethics review body unaffiliated with the author and SUNBLAZE Co., Ltd.

### Measurement instruments

Personality traits were measured using the Japanese version of the HEXACO-60 (Wakabayashi, 2014), which consists of 60 items assessing six domains: Honesty–Humility, Emotionality, Extraversion, Agreeableness, Conscientiousness, and Openness. Items are rated on a 5-point Likert scale ranging from 1 (strongly disagree) to 5 (strongly agree). In the present sample, internal consistency coefficients were *α* = 0.571 for Honesty–Humility, α = 0.830 for Emotionality, *α* = 0.621 for Extraversion, *α* = 0.783 for Agreeableness, *α* = 0.815 for Conscientiousness, and α = 0.804 for Openness.

The Dark Triad traits were assessed using the Japanese version of the Short Dark Triad (SD3-J; Shimotsukasa and Oshio, 2017), consisting of 27 items (9 items each for Machiavellianism, Narcissism, and Psychopathy), rated on a 5-point Likert scale. In the present sample, internal consistency coefficients were α = 0.767 for Machiavellianism, α = 0.778 for Narcissism, and α = 0.708 for Psychopathy, with α = 0.842 for the SD3-J total score.

Power harassment was assessed using the Workplace Power Harassment Scale developed by Tou et al. (2017). The scale comprises 18 items loading on three factors: power-harassing behaviors (12 items), power-harassment climate (4 items), and power-harassing attitudes (2 items). Following Tou et al. (2017), the “climate” factor here is supervisor-centered (subordinate constriction and silence around the supervisor) rather than a general organizational climate; the attitude factor reflects a domineering and non-receptive stance that can underlie and legitimize coercive supervision; and the original validation supported the three-factor structure (CFI = 0.962, RMSEA = 0.074). Participants responded with reference to their actual workplace, indicating the extent to which (a) they had engaged in the listed behaviors, (b) their interpersonal context was characterized by subordinate constriction and silence, and (c) they endorsed the attitude items, on a 3-point scale (1 = not applicable, 2 = applicable once, 3 = applicable multiple times). Internal consistency in the present sample was α = 0.862 (behaviors), α = 0.730 (climate), α = 0.760 (attitudes), and α = 0.880 (total). Importantly, this composite combines three conceptually distinct components—a behavioral perpetration component, an attitudinal endorsement component, and an interpersonal-climate component reflecting subordinate reactions—into a single perpetration-tendency index. We acknowledge this criterion heterogeneity explicitly: the composite is not a pure behavioral-frequency measure, and substantively it is best read as a perpetration-related composite that bundles behavior, attitude, and supervisor-centered interpersonal dynamics. Because the publicly archived dataset (see Data Availability) retains only the composite total—not the 18 individual items—subscale-level multiple regressions cannot be reproduced from the released file by external readers, although the conceptual implications of the composite structure are revisited in the Limitations. The item-level limitations are also revisited in connection with the sample composition: the items presuppose a relational context in which the respondent occupies a position with subordinates, and managerial status was not separately assessed (see Limitations).

Gender harassment was assessed using the Gender Harassment Scale developed by Kobayashi and Tanaka (2010). The scale consists of 13 items and a two-factor structure reflecting (a) gender-harassing commission behaviors and (b) gender-harassing omission behaviors in workplace contexts. Commission items capture behaviors that impose gender-role expectations on women, whereas omission items reflect withholding opportunities or expectations based on gender. Responses were rated on a 5-point frequency scale (1 = never engaged in the behavior, 5 = frequently engaged in the behavior). In the original validation study, internal consistency coefficients were reported as *α* = 0.815 for the commission subscale and α = 0.868 for the omission subscale, with similar estimates reported in subsequent samples (α = 0.848 and 0.845, respectively). In the present sample, internal consistency coefficients were α = 0.876 for commission behaviors, α = 0.812 for omission behaviors, and α = 0.901 for the total gender harassment index.

For the primary regression analyses, the harassment subscales were aggregated into a power-harassment composite and a gender-harassment composite, in line with the conceptual focus of the present study on overall perpetration tendencies and in light of the theoretical and empirical overlap among the subscales. Aggregation also reduced model complexity and avoided parameter inflation in multivariate regressions. We acknowledge, however, that the power-harassment composite is criterion-heterogeneous: it bundles behavioral enactment, attitudinal endorsement, and supervisor-centered interpersonal-climate items into one index (see Measurement Instruments). Findings should accordingly be read as relating to a perpetration-related composite, not to behavioral perpetration in a narrow sense; this caveat is revisited in the Limitations.

### Procedure

The survey was conducted from August 1 to August 18, 2025. The order of responses was HEXACO, SD3-J, Power Harassment, and Gender Harassment. Participants received Amazon gift cards as compensation. Participants were instructed to respond to the harassment items from the perspective of their own behaviors in the workplace. Specifically, they were asked to indicate how frequently they themselves had engaged in the described behaviors within their current or recent workplace context. Thus, the present study assessed harassment perpetration tendencies rather than victimization experiences.

### Data processing

Because every Google Forms item was configured as required by the researcher, item-level missingness from skipped items did not occur; the small number of technical missing values (≤ 0.3% per indicator) was handled by complete-case analysis. Continuous variables included in regression analyses were z-standardized (mean = 0, SD = 1) so that regression slopes are interpretable as standardized *β*. Gender was coded as a binary indicator (0 = male, 1 = female). Geographic region was self-reported and, in line with the de-identification of the public dataset, was retained at the level of three categories—Kanto, Kinki, and Other—with Kanto as the reference category, yielding two dummy indicators (Kinki vs. Kanto; Other vs. Kanto). This covariate was included to absorb potential confounding due to regional differences in occupational composition and workplace norms. Distributional and influence diagnostics were inspected as part of the regression workflow: residual normality (Shapiro–Wilk), residual autocorrelation (Durbin–Watson), heteroscedasticity (Breusch–Pagan), and high-leverage cases (Cook’s distance with a 4/n threshold). Multicollinearity was assessed with the Variance Inflation Factor (VIF), with general cutoffs of 5–10.

### Analytical strategy

All analyses were conducted in Python (pandas, statsmodels, SciPy). We first computed Spearman rank correlations among all focal variables to accommodate skewed distributions and potential outliers. For prediction, we estimated a series of multiple regression models with HC3 robust standard errors. Effect sizes were reported as standardized coefficients (β), and statistical significance was evaluated using two-tailed tests at conventional levels.

Sample size consideration (*post hoc* sensitivity). Because an *a priori* power analysis was not conducted at the design stage, we performed a post hoc sensitivity analysis focusing on the incremental explanatory power of HEXACO traits beyond covariates and Dark Triad traits (i.e., ΔR^2^ from Model A to Model B). With *N* = 353 and six HEXACO predictors, the observed incremental effect corresponded to Cohen’s f^2^ = 0.040 (ΔR^2^ = 0.032) for power harassment, yielding achieved power = 0.803 at *α* = 0.05. For gender harassment, the incremental effect was f^2^ = 0.122 (ΔR^2^ = 0.096), yielding achieved power = 0.9997 at α = 0.05.

#### Model definitions

Three hierarchical regression models were estimated. Model A included only control variables (age, gender, and region) together with the three Dark Triad traits—Machiavellianism, Narcissism, and Psychopathy. Model B extended Model A by adding the six HEXACO personality factors to examine their incremental contribution. Finally, Model C further incorporated the interaction terms between Honesty–Humility (H–H) and each of the Dark Triad traits to test potential moderating effects (Equations 1-3).


$$\begin{array}{c} \text{Model}\;A=\text{controls}\;(\mathrm{age},\text{gender},\text{region})\\ +\text{Dark Triad}\;(\text{Machiavellianism},\text{Narcissism},\text{Psychopathy}) \end{array}$$ (1)

ModelB=ModelA+HEXACO(sixfactors) (2)

$$\begin{array}{c} \text{Model}\;C=\text{Model}\;B+\text{interactions between Honesty}\\ –\text{Humility}\;(H–H)\;\text{and each Dark Triad trait} \end{array}$$ (3)

#### Primary inference and incremental validity

Harassment perpetration tendencies (power harassment, gender harassment) were the primary outcomes. We emphasize Model B coefficients for substantive interpretation (see Table 1: B_controls+DT+HEXACO), and we evaluate the added value of HEXACO via Δ*R*^2^ = *R*^2^(B) − *R*^2^(A) and corresponding F-tests (see Table 2). To probe moderation, Model C added H–H × Dark Triad interactions; when incremental fit over Model B was negligible, we report this succinctly (see Table 2).

**Table 1:** Descriptive statistics for HEXACO-60 subscales, SD3-J subscales, harassment composite scores, and demographic variables (*N* = 354).

Regression coefficients					
Dependent variable	Model	Predictor	β	SE (robust)	t value
Power harassment	A_controls+DT	Machiavellianism	−0.06	0.06	−0.95
		Narcissism	0	0.06	−0.08
		Psychopathy	0.4***	0.06	6.65
	B_controls+DT+HEXACO	Machiavellianism	−0.11	0.07	−1.52
		Narcissism	0	0.08	0.05
		Psychopathy	0.32***	0.06	4.89
		Honesty-Humility	−0.14*	0.07	−1.97
		Emotionality	−0.03	0.06	−0.54
		Extraversion	−0.06	0.07	−0.81
		Agreeableness	−0.11	0.06	−1.86
		Conscientiousness	−0.05	0.06	−0.91
		Openness	0.03	0.05	0.60
	B_sensitivity_(drop_high_Cooks)	Machiavellianism	−0.06	0.06	−0.98
		Narcissism	0.06	0.08	0.86
		Psychopathy	0.26***	0.06	4.31
		Honesty-Humility	−0.09	0.07	−1.24
		Emotionality	−0.06	0.06	−1.02
		Extraversion	−0.1	0.06	−1.65
		Agreeableness	−0.17**	0.06	−2.88
		Conscientiousness	−0.05	0.05	−0.96
		Openness	−0.04	0.06	−0.69
	C_controls+DT+HEXACO+DTxHH	Machiavellianism	−0.11	0.07	−1.52
		Narcissism	0.02	0.08	0.19
		Psychopathy	0.3***	0.06	4.67
		Honesty-Humility	−0.14	0.07	−1.95
		Emotionality	−0.01	0.05	−0.24
		Extraversion	−0.06	0.07	−0.89
		Agreeableness	−0.11	0.06	−1.81
		Conscientiousness	−0.04	0.06	−0.59
		Openness	0.05	0.06	0.87
	B_by_gender	Machiavellianism	−0.17	0.11	−1.52
		Narcissism	0.22	0.14	1.57
		Psychopathy	0.45***	0.12	3.67
		Honesty-Humility	−0.01	0.14	−0.07
		Emotionality	0.06	0.09	0.67
		Extraversion	−0.11	0.11	−0.97
		Agreeableness	−0.02	0.10	−0.20
		Conscientiousness	0	0.08	−0.01
		Openness	−0.12	0.13	−0.93
		Machiavellianism	−0.08	0.11	−0.72
		Narcissism	−0.09	0.10	−0.88
		Psychopathy	0.24**	0.08	2.93
		Honesty-Humility	−0.19*	0.10	−1.98
		Emotionality	−0.07	0.07	−0.99
		Extraversion	−0.01	0.09	−0.14
		Agreeableness	−0.17*	0.07	−2.33
		Conscientiousness	−0.07	0.08	−0.83
		Openness	0.1	0.06	1.60
Gender harassment	A_controls+DT	Machiavellianism	−0.14**	0.05	−2.73
		Narcissism	0.18***	0.05	3.42
		Psychopathy	0.16**	0.06	2.85
	B_controls+DT+HEXACO	Machiavellianism	−0.16**	0.06	−2.96
		Narcissism	0.19**	0.07	2.86
		Psychopathy	0.15**	0.06	2.62
		Honesty-Humility	−0.23***	0.07	−3.52
		Emotionality	0.03	0.05	0.63
		Extraversion	−0.03	0.06	−0.49
		Agreeableness	0.1	0.05	1.89
		Conscientiousness	0.03	0.05	0.65
		Openness	−0.24***	0.06	−4.20
	B_sensitivity_(drop_high_Cooks)	Machiavellianism	−0.17**	0.05	−3.27
		Narcissism	0.17**	0.06	2.69
		Psychopathy	0.14*	0.06	2.28
		Honesty-Humility	−0.23***	0.06	−3.72
		Emotionality	0.04	0.05	0.72
		Extraversion	−0.03	0.07	−0.51
		Agreeableness	0.11*	0.05	1.99
		Conscientiousness	0.02	0.05	0.42
		Openness	−0.24***	0.06	−4.37
	C_controls+DT+HEXACO+DTxHH	Machiavellianism	−0.16**	0.06	−2.86
		Narcissism	0.18**	0.07	2.74
		Psychopathy	0.15**	0.06	2.60
		Honesty-Humility	−0.22***	0.06	−3.51
		Emotionality	0.04	0.05	0.78
		Extraversion	−0.03	0.06	−0.50
		Agreeableness	0.11*	0.05	1.98
		Conscientiousness	0.03	0.05	0.65
		Openness	−0.23***	0.06	−3.92
	B_by_gender	Machiavellianism	−0.11	0.10	−1.09
		Narcissism	0.22	0.14	1.58
		Psychopathy	0.22*	0.11	1.96
		Honesty-Humility	−0.13	0.14	−0.97
		Emotionality	−0.03	0.09	−0.37
		Extraversion	0.08	0.12	0.62
		Agreeableness	0.09	0.10	0.86
		Conscientiousness	0.08	0.08	0.99
		Openness	−0.44***	0.12	−3.84
		Machiavellianism	−0.21**	0.08	−2.71
		Narcissism	0.16	0.08	1.94
		Psychopathy	0.12	0.07	1.76
		Honesty-Humility	−0.27**	0.09	−3.21
		Emotionality	0.08	0.06	1.37
		Extraversion	−0.07	0.08	−0.93
		Agreeableness	0.08	0.07	1.18
		Conscientiousness	0.01	0.06	0.11
		Openness	−0.13	0.07	−1.87

Model B = controls (age, gender, region) + Dark Triad (M, N, P) + HEXACO (H–H, E, X, A, C, O). Internal labels: B_controls+DT+HEXACO = Model B (overall); B_by_gender = Model B estimated separately by gender. Standardized β; HC3 robust SE; two-tailed tests; ***p < 0.001, **p < 0.01, *p < 0.05. Bold values indicate the principal summary metric.

**Table 2:** Spearman rank correlations among HEXACO factors, Dark Triad traits, and harassment perpetration tendencies.

Incremental model fit and diagnostic statistics							
Dependent variable	R^2^ (model A)	R^2^ (model B)	ΔR^2^ (B − A)	FΔ (B − A)	pΔ	R^2^ (B, sensitivity)	R^2^ (C)
Power harassment	0.166	0.198	0.032	2.280	0.036	0.221	0.218
Gender harassment	0.117	0.213	0.096	6.910	0.000	0.203	0.219

Dependent variable	Durbin–Watson (model B)	Breusch–Pagan LM p	Breusch–Pagan F p	Max Cook’sD (Model B)	N (Cook’s D > 4/n, Model B)
Power harassment	1.95	0.00	0.00	0.06	23
Gender harassment	1.84	0.00	0.00	0.04	15

Model A = controls (age, gender, and region [Kinki vs. other regions; reference = other regions]) + Dark Triad traits. Model B = Model A + HEXACO traits. Model C = Model B + Dark Triad × Honesty–Humility interaction terms. Cook’s D values are reported for Model B. Cases with Cook’s D > 4/n were considered high-influence observations. Durbin–Watson statistics assess residual autocorrelation. Breusch–Pagan tests assess heteroskedasticity.

#### Sensitivity and stratified analyses

As a sensitivity analysis, we re-estimated Model B after excluding high-influence observations using Cook’s distance > 4/n (see Table 2). For diagnostics, see Table 3; VIFs are in Table 4. For gender-stratified evidence, we fit Model B separately for men and women and compare explained variance and key coefficients (see Table 5; coefficients also summarized in Table 1: B_by_gender).

**Table 3:** HC3-robust standardized regression coefficients (β, robust SE, t, p) for Model A (controls + Dark Triad), Model B (Model A + HEXACO), Model B sensitivity (excluding cases with Cook’s D > 4/n), and Model C (Model B + Honesty–Humility × Dark Triad interactions).

Model diagnostics					
Dependent variable	DW	Shapiro–Wilk p	Breusch-Pagan p	Max Cook’s D	N Cook’s D > 4/n
Power harassment	1.95	2.14E-14	0.00	0.06	23
Gender harassment	1.84	6.30E-09	3.53E-06	0.04	15

**Table 4:** Model fit and incremental validity statistics (R², adjusted R², ΔR², F-change, p-change, Cohen’s f², achieved power) and diagnostic statistics for Model B.

Variance inflation factors (VIF)		
Dependent variable	Predictor	VIF
Power harassment	Age	1.24
	Gender	1.11
	area_simplified_Kinki	1.17
	area_simplified_Other	1.16
	Machiavellianism	1.31
	Narcissism	2.00
	Psychopathy	1.56
	Honesty-Humility	1.57
	Emotionality	1.33
	Extraversion	1.70
	Agreeableness	1.53
	Conscientiousness	1.16
	Openness	1.31
Gender harassment	AGE	1.24
	gender	1.11
	area_simplified_Kinki	1.17
	area_simplified_Other	1.16
	Machiavellianism	1.31
	Narcissism	2.00
	Psychopathy	1.56
	Honesty-Humility	1.57
	Emotionality	1.33
	Extraversion	1.70
	Agreeableness	1.53
	Conscientiousness	1.16
	Openness	1.31

**Table 5:** Sex-stratified Model B explained variance (R², adjusted R²) for power-harassment and gender-harassment perpetration tendencies, separately for men (*n* = 133) and women (*n* = 220).

Gender-stratified model fit				
Dependent variable	Gender	*N*	R^2^	Adj. R^2^
Power harassment	0 = Male	133	0.29	0.22
	1 = Female	220	0.20	0.16
Gender harassment	0 = Male	133	0.28	0.21
	1 = Female	220	0.17	0.12

#### Diagnostics and multicollinearity

We inspected distributional and heteroskedasticity diagnostics and relied on HC3 for inference. Multicollinearity was assessed via variance inflation factors; VIF results are reported in Table 4.

### Summary of model diagnostics

Model diagnostics included checks of residual normality (Shapiro–Wilk test), autocorrelation (Durbin–Watson statistic), heteroscedasticity (Breusch–Pagan test), and influential cases (Cook’s distance, with a threshold of 4/n). Multicollinearity was assessed using variance inflation factors (VIF), with commonly used cutoffs (5–10). Robust standard errors were adopted to address violations of normality or heteroscedasticity, and sensitivity analyses were conducted to ensure the robustness of the results. Final models were selected with an emphasis on interpretability.

### Software and reproducibility

Analyses were conducted with Python 3.12.3. Reproducible scripts and model outputs (correlation tables, regression coefficients, diagnostic statistics, VIF lists) can be provided as appendices or in a repository, with access conditions specified in the Data Availability section. No procedures requiring random seeds were employed.

## Results

### Descriptive statistics

Table 6 presents descriptive statistics for the primary variables. Mean scores (SDs) for the HEXACO factors were Honesty–Humility (H–H) 3.25 (0.59), Emotionality (E) 3.30 (0.78), Extraversion (X) 2.70 (0.84), Agreeableness (A) 2.88 (0.73), Conscientiousness (C) 3.21 (0.68), and Openness (O) 3.46 (0.80). For the Dark Triad, means (SDs) were Machiavellianism 3.58 (0.66), Narcissism 2.40 (0.72), and Psychopathy 2.50 (0.66). Harassment perpetration tendencies averaged 1.26 (0.32) for power harassment and 1.70 (0.70) for gender harassment. The sample spans a broad age range and includes adequate numbers of both men and women.

**Table 6:** Summary of regression diagnostic statistics for Model B (Durbin–Watson, Shapiro–Wilk, Breusch–Pagan).

Descriptive statistics							
Variable	*N*	M	SD	min	max	sk	ku
1. Honesty-humility	354	3.25	0.59	1.5	5.0	−0.18	0.42
2. Emotionality	354	3.30	0.78	1.1	5.0	−0.39	0.01
3. Extraversion	354	2.70	0.84	1.0	4.8	0.25	−0.40
4. Agreeableness	354	2.88	0.73	1.0	4.8	−0.11	−0.07
5. Conscientiousness	354	3.21	0.68	1.3	4.9	0.15	−0.10
6. Openness	354	3.46	0.80	1.4	5.0	−0.11	−0.62
7. Machiavellianism	353	3.58	0.66	1.1	4.9	−0.46	0.24
8. Narcissism	353	2.40	0.72	1.0	4.9	0.34	−0.06
9. Psychopathy	353	2.50	0.66	1.1	4.4	0.13	−0.40
10. Power harassment	354	1.26	0.32	1.0	2.5	1.81	3.04
11. Gender harassment	354	1.70	0.70	1.0	4.2	1.14	0.65
12. Age	354	29.10	11.13	10.0	60.0	0.35	−0.09
13. Gender	354	0.62	0.49	0.0	1.0	−0.50	−1.76

Gender was coded as 0 = male and 1 = female; thus, the mean reflects the proportion of participants coded as 1 (female). sk = skewness; ku = kurtosis.

### Correlation analysis

Spearman rank correlations among HEXACO, Dark Triad traits, and harassment perpetration tendencies are shown in Table 7. H–H correlated negatively with Machiavellianism (*ρ* = −0.32, *p* < 0.001), Narcissism (ρ = −0.36, *p* < 0.001), and Psychopathy (ρ = −0.38, *p* < 0.001), and also with power-harassment perpetration tendencies (ρ = −0.23, *p* < 0.001) and gender-harassment perpetration tendencies (ρ = −0.18, *p* < 0.001). Agreeableness correlated negatively with Machiavellianism (ρ = −0.27, *p* < 0.001), Psychopathy (ρ = −0.36, *p* < 0.001), and power-harassment perpetration tendencies (ρ = −0.26, *p* < 0.001). By contrast, Extraversion correlated positively with Narcissism (ρ = +0.56, *p* < 0.001), Psychopathy (ρ = +0.16, *p* < 0.01), and gender-harassment perpetration tendencies (ρ = +0.11, *p* < 0.05). Machiavellianism’s bivariate associations with harassment outcomes were small and in opposite directions: a small positive association with power-harassment perpetration tendencies (ρ = +0.12, *p* < 0.05) and a small negative association with gender-harassment perpetration tendencies (ρ = −0.10, *p* ≈ 0.055). These bivariate patterns are consistent with the multiple-regression results reported below: Honesty–Humility and Agreeableness are negatively associated with harassment outcomes at the bivariate level, the negative regression coefficient for Machiavellianism in adjusted gender-harassment models is consistent in sign with its small bivariate negative correlation rather than emerging via sign reversal under adjustment, and Extraversion’s positive bivariate associations with narcissism and psychopathy underscore that interpersonal assertiveness can co-occur with antagonistic content within the present sample.

**Table 7:** Variance inflation factors (VIF) for Model B predictors.

Correlation matrix										
Variable	2	3	4	5	6	7	8	9	10	11
1. Honesty-Humility	0.04	−0.19***	0.24***	0.15**	−0.03	−0.32***	−0.36***	−0.38***	−0.23***	−0.18***
2. Emotionality	1	−0.33***	−0.30***	−0.1	−0.26***	0.04	−0.32***	−0.09	−0.02	−0.01
3. Extraversion		1	0.23***	0.08	0.24***	−0.09	0.56***	0.16**	−0.05	0.11*
4. Agreeableness			1	0.03	0.05	−0.27***	0.02	−0.36***	−0.26***	0.01
5. Conscientiousness				1	0.18***	0.07	0.13*	−0.09	−0.11*	−0.06
6. Openness					1	0.14**	0.27***	0.08	0.06	−0.20***
7. Machiavellianism						1	0.14**	0.29***	0.12*	−0.1
8. Narcissism							1	0.39***	0.12*	0.21***
9. Psychopathy								1	0.34***	0.15**
10. Power harassment									1	0.35***
11. Gender harassment										1

***p < 0.001, **p < 0.01, *p < 0.05. Bold values indicate the principal summary metric.

### Multiple regression analysis

We conducted hierarchical regressions predicting power and gender harassment. Model A included controls (age, gender, region) and the Dark Triad; Model B additionally included the six HEXACO factors. Below we report standardized *β* with HC3-robust *p*-values (coefficients = Table 1; model comparison = Table 2). As a robustness check, we also evaluated whether the regression estimates were disproportionately driven by a small number of highly influential observations. Because extreme cases may reflect substantively meaningful heterogeneity as well as undue statistical leverage, we used Cook’s distance to identify high-influence cases and re-estimated the models after excluding observations exceeding a conventional influence threshold; the substantive conclusions were unchanged (Table 2). For interpretation, we treated standardized coefficients as descriptive indicators of association strength rather than applying strict cutoffs. For incremental validity, we interpreted ΔR^2^ in light of conventional effect-size guidelines for explained variance, such as Cohen’s f^2^ (where f^2^ ≈ 0.02, 0.15, and 0.35 are often described as small, medium, and large, respectively) (Cohen, 1988). Accordingly, modest increments in explained variance (e.g., ΔR^2^ on the order of 0.02–0.03) may still be practically meaningful depending on the outcome and context.

Power harassment. In Model A, only Psychopathy was significant (*β* = 0.396, *p* < 0.001), whereas the other Dark Triad traits were not (M: *β* = −0.062, *p* = 0.342; *N*: *β* = −0.005, *p* = 0.933). In Model B, R^2^ increased from 0.166 to 0.198, with ΔR^2^ = 0.032, F change = 2.28, *p* = 0.036. Key predictors in Model B were Psychopathy (*β* = 0.317, *p* < 0.001) and H–H (*β* = −0.143, *p* = 0.049); Agreeableness was marginal (*β* = −0.108, *p* = 0.063) (Tables 1, 2).

Gender harassment. In Model A, Machiavellianism (β = −0.138, *p* = 0.006), Narcissism (β = +0.180, *p* < 0.001), and Psychopathy (β = +0.165, *p* = 0.004) were each significant. In Model B, R^2^ rose from 0.117 to 0.213, with ΔR^2^ = 0.096, F change = 6.91, *p* < 0.001. H–H (β = −0.230, *p* < 0.001) and Openness (β = −0.236, *p* < 0.001) were negative predictors, while the Dark Triad effects remained significant in adjusted form (M: *β* = −0.164, *p* = 0.003; N: β = +0.190, *p* = 0.004; P: β = +0.154, *p* = 0.009). Gender was also significant (β = −0.312, *p* = 0.006; coded 0 = male, 1 = female; the negative sign indicates higher reported gender-harassment perpetration tendencies among men). We note explicitly that the negative coefficient on Machiavellianism in Model B is not a sign reversal: Machiavellianism’s bivariate correlation with gender-harassment perpetration tendencies in this sample was already small and negative (*ρ* ≈ −0.10; see Correlation Analysis). Adjustment for the other Dark Triad components (narcissism, psychopathy) and for the HEXACO factors then amplifies, rather than introduces, this negative coefficient, by partialling out variance in Machiavellianism that is shared with the other antagonistic predictors. We therefore do not interpret the negative coefficient as evidence that Machiavellianism is ‘protective’ against gender-harassment perpetration tendencies. Three more parsimonious accounts are simultaneously plausible and cannot be discriminated by the present design: (i) shared antagonistic variance with narcissism and psychopathy is partialled out, leaving the residual Machiavellianism variance to absorb other content (a magnitude-amplification form of suppression, not a sign reversal); (ii) substantial predictor overlap among Dark Triad components produces a partialling-style coefficient pattern in OLS; and (iii) model instability with three correlated antagonistic predictors. We therefore present this coefficient descriptively, as a feature of the multivariate solution rather than as a substantive trait-level finding.

### Sensitivity analysis and interaction effects

Re-estimating Model B after removing cases with Cook’s distance > 4/n yielded R^2^ B sensitivity of 0.221 (power harassment) and 0.203 (gender harassment), indicating that main findings were largely preserved (details = Table 2; max Cook’s D = 0.058/0.043; exceedances = 23/15). Adding H–H × M/N/P interactions produced R^2^ with interactions of 0.218 (power) and 0.219 (gender), representing only small improvements over Model B (Table 2).

### Gender-stratified analyses

When Model B was estimated separately for men (*n* = 133) and women (*n* = 220), the proportion of explained variance and the predictor structure differed between sexes (Tables 5, 8). For power-harassment perpetration tendencies, R^2^ was 0.287 among men and 0.203 among women. Among men, the only HC3-significant trait predictor was Psychopathy (*β* = +0.45, *p* < 0.001); H–H, Agreeableness, and the other HEXACO factors were not significant. Among women, Psychopathy retained a significant positive association (β = +0.24, *p* = 0.003), and in addition H–H (β = −0.19, *p* = 0.047) and Agreeableness (β = −0.17, *p* = 0.020) were negatively associated with power-harassment perpetration tendencies. For gender-harassment perpetration tendencies, R^2^ was 0.283 among men and 0.166 among women. Among men, Openness was the dominant negative predictor (β = −0.44, *p* < 0.001), while Psychopathy was marginal (β = +0.22, *p* = 0.050) and the other dark traits and HEXACO factors were not significant. Among women, by contrast, H–H was the strongest negative predictor (β = −0.27, *p* = 0.001) and Machiavellianism showed the same conditional negative coefficient described above (β = −0.21, *p* = 0.007), with narcissism marginal (β = +0.16, *p* = 0.053). These sex-stratified patterns are consistent with the hypothesis (H5) that the relative weights of HEXACO and Dark-Triad traits in adjusted models depend on respondent sex—particularly for gender-harassment perpetration tendencies—rather than reflecting a single homogeneous psychological structure across sexes. We caution that differences in *n* and outcome variance by sex limit precision of the female-specific (and especially the male-specific) estimates, and that the present design does not formally test the equality of regression coefficients across sex (such formal tests would require multi-group SEM or interaction terms across the full sample with sex).

**Table 8:** Sex-stratified model B coefficients (HC3-robust standardized β).

Variable	Power harassment Men (*n* = 133)	Power harassment Women (*n* = 220)	Gender harassment Men (*n* = 133)	Gender harassment Women (*n* = 220)
Age	−0.069	−0.057	+0.010	+0.158*
Area: Kinki vs. Kanto	−0.304	+0.108	−0.013	−0.030
Area: Other vs. Kanto	−0.015	−0.008	−0.244	−0.144
Machiavellianism	−0.172	−0.076	−0.109	−0.213**
Narcissism	+0.224	−0.085	+0.224	+0.159†
Psychopathy	+0.447***	+0.237**	+0.217*	+0.120†
Honesty–Humility	−0.010	−0.191*	−0.131	−0.274**
Emotionality	+0.060	−0.069	−0.032	+0.080
Extraversion	−0.106	−0.012	+0.077	−0.071
Agreeableness	−0.019	−0.174*	+0.087	+0.081
Conscientiousness	−0.001	−0.068	+0.083	+0.007
Openness	−0.119	+0.100	−0.442***	−0.125†

Each column reports HC3-robust standardized β coefficients from a sex-stratified Model B (controls + Dark Triad + HEXACO) estimated separately for men (gender = 0) and women (gender = 1). Significance markers: † *p* < 0.10; * *p* < 0.05; ** *p* < 0.01; *** *p* < 0.001. The “Gender (1 = female)” predictor is not estimated within sex-stratified models. Formal tests of cross-sex equality of regression coefficients (e.g., multi-group SEM) are not estimated here; the divergence in male versus female estimates should be read as a hypothesis-generating pattern, not as a confirmed moderation effect.

### Model diagnostics

Durbin–Watson statistics were 1.95 (power) and 1.84 (gender), suggesting little residual autocorrelation. Shapiro–Wilk and Breusch–Pagan tests were significant, indicating deviations from normality and homoscedasticity; inference therefore relies on HC3 robust SEs (Table 3). VIFs were modest (max 2.00 for N; mean 1.40), implying no serious multicollinearity (top values: *N* = 2.00, X = 1.70, H–H = 1.57; Table 4). Missingness was minimal (each Dark Triad variable 0.28%; others 0%), yielding an effective sample of *n* = 353.

## Discussion

### Summary of results

This study examined the associations among HEXACO traits, Dark Triad traits, and self-reported workplace harassment perpetration tendencies in a sample of currently employed Japanese adults, and additionally examined whether the predictor structure differed by sex. Three patterns are most relevant for the hypotheses outlined in the Purpose. First, in adjusted models psychopathy retained a positive association with power-harassment perpetration tendencies (consistent with H1) and was a stronger adjusted predictor of power-harassment perpetration than of gender-harassment perpetration tendencies, particularly among men (consistent with H2). Second, the HEXACO factors yielded a non-trivial increase in explained variance over the Dark Triad and covariates (ΔR^2^ = 0.032 for power, 0.096 for gender; consistent with H3), with H–H and (for gender harassment) Openness in the negative direction (consistent with H4 for H–H; partially supportive of H4 for Agreeableness, which was negative but only marginal in the full sample). Third, sex-stratified analyses revealed a marked divergence in predictor structure (consistent with H5): for gender-harassment perpetration tendencies, Openness dominated as a negative predictor among men, whereas H–H and (conditionally) Machiavellianism dominated among women. We do not interpret the Machiavellianism sign reversal in adjusted models as substantive “protection” but as a feature of multivariate estimation under correlated antagonistic predictors. Likewise, we describe H–H’s adjusted negative coefficient as an adjusted association rather than as evidence that the residual H–H variance reflects a separable latent fairness/sincerity factor distinct from Dark-Triad antagonism.

### Theoretical implications

The present findings are broadly consistent with prior research linking HEXACO and the Dark Triad to socially aversive workplace behavior, while also pointing to two more specific patterns. First, at the full-sample level, H–H showed a consistent negative adjusted association across both harassment outcomes, and adding the HEXACO factors increased the explained variance over the Dark Triad alone. Two interpretive caveats are essential here. (a) The variance in H–H that remains after adjusting for the Dark Triad is a residualized observed-score component, not a separable latent factor; the present analyses do not estimate a latent-variable model that would be required to claim that this residual specifically indexes a fairness/sincerity disposition distinct from antagonism. We therefore frame the H–H finding as ‘adjusted association’ rather than as evidence of moral-personality specificity. (b) Machiavellianism’s negative coefficient in adjusted models for gender-harassment perpetration tendencies is consistent in sign with its small bivariate negative correlation in this sample (*ρ* ≈ −0.10), not a sign reversal that emerges only under adjustment. Adjustment for the other Dark Triad components and for the HEXACO factors amplifies the magnitude of this negative coefficient by partialling out shared antagonistic variance, but it does not flip its sign. We therefore do not develop a theoretical account that depends on Machiavellianism being protective; we read the adjusted coefficient as a feature of multivariate estimation under correlated antagonistic predictors rather than as a substantive trait-level effect. Second, the sex-stratified results add substantive structure that is not visible in the pooled estimates. For gender-harassment perpetration tendencies, the dominant negative predictor among men was Openness, while among women it was H–H. Mean-level sex differences in HEXACO domains (notably the elevation of women on Emotionality, with smaller differences on the other domains) are well documented (Costa et al., 2001; Schmitt et al., 2008), but the present finding concerns trait–outcome associations within each sex rather than mean-level differences. One reading—offered cautiously and not as a confirmed mechanism—is that men’s gender-harassment perpetration tendencies in this sample are more closely linked to a low-Openness configuration that may track rigid, conventional gender-role schemas, while women’s gender-harassment perpetration tendencies are more closely linked to a low H–H configuration consistent with low fairness/non-exploitation. We emphasize that this is one provisional interpretation among several, that the male and female estimates differ in precision (*n* = 133 vs. 220), and that we do not formally test the cross-sex equality of regression coefficients. Finally, the personality–harassment associations observed in this Japanese employee sample are generally in line with patterns reported in Western contexts; however, the present study does not permit direct conclusions about cultural universality. Direct cross-cultural comparisons using harmonized measures and matched occupational samples remain a priority for future work.

To situate the present findings within the broader empirical literature, we compare our key effect sizes and patterns with those reported in prior meta-analyses and primary studies. First, the adjusted psychopathy–power-harassment association in this study (*β* ≈ 0.32–0.40) exceeds the meta-analytic corrected correlation between psychopathy and counterproductive work behavior reported by O'Boyle et al. (2012; r̄ = 0.19 for CWB-organizational) and is comparable in magnitude to the psychopathy–abusive-supervision associations reviewed by LeBreton et al. (2018) and Boddy (2011). This difference likely reflects the fact that power-harassment perpetration, as operationalized by Tou et al. (2017), is a more targeted supervisor-centered construct than general CWB, yielding stronger associations with the callous-impulsive core of psychopathy. Our result replicates this pattern in a Japanese employee sample, extending the cross-cultural evidence base beyond the Western contexts that dominate the meta-analytic literature. Second, the negative adjusted association between Honesty–Humility and harassment perpetration tendencies in this study (β ≈ −0.14 for power, −0.23 for gender) is broadly consistent with the meta-analytic domain-level correlation between H–H and CWB reported by Pletzer et al. (2019a; r̄ ≈ −0.26), and with Lee et al. (2019), who identified H–H as the single most informative HEXACO factor for workplace deviance. The incremental variance explained by the HEXACO factors over the Dark Triad and demographic covariates (ΔR^2^ = 0.032 for power, 0.096 for gender) has no direct meta-analytic benchmark because prior work has typically tested HEXACO increments over the Big Five rather than over the Dark Triad; however, the ΔR^2^ values observed here fall within the range of incremental effects reported in Pletzer et al. (2019a) for HEXACO over Big Five in predicting CWB (ΔR^2^ ≈ 0.02–0.08 across studies), suggesting comparable explanatory gain. Third, the negative Machiavellianism coefficient in adjusted gender-harassment models is consistent with this sample’s bivariate negative correlation (*ρ* ≈ −0.10) and may reflect the high inter-trait overlap among Dark Triad components documented in the Vize et al. (2018) meta-analysis, which showed Machiavellianism shares the most variance with narcissism and psychopathy. Under simultaneous adjustment, residual Machiavellianism variance may absorb content distinct from the shared antagonistic core, a phenomenon also noted in Western samples (Jones and Paulhus, 2009). Whether the negative bivariate sign itself is culturally specific (e.g., reflecting strategic restraint in gendered contexts within Japanese organizations) or a sampling artifact remains to be tested in direct cross-cultural comparisons. Fourth, the sex-stratified divergence in predictor structure — Openness as the dominant negative predictor of gender-harassment perpetration tendencies among men, versus H–H among women — has no direct precedent in the literature, making it a novel contribution. Cross-cultural meta-analyses have documented mean-level sex differences in HEXACO domains (Costa et al., 2001; Schmitt et al., 2008), but within-sex trait–outcome associations have rarely been examined for harassment perpetration, and no prior study has reported the Openness pattern observed here for men. Finally, comparisons with prior Japanese Dark Triad research (Shimotsukasa and Oshio, 2017, 2019; Koshinaka, 2020; Oda and Matsumoto-Oda, 2022) show that the present study extends the domestic evidence base from manipulative strategies, interpersonal aversion, and prosociality into workplace harassment perpetration specifically — a domain that had remained largely unexamined in Japanese samples despite its public-health relevance (Tsuno et al., 2015).

### Practical implications

Although the present study was cross-sectional and based on self-report, the findings provide several tentative implications for workplace-harassment prevention and personnel management—offered as hypotheses for further work rather than as endorsements of personality-based screening. The observed adjusted associations are consistent with the broader literature linking lower Honesty–Humility and Agreeableness, and higher Dark Triad traits, with elevated self-reported harassment perpetration tendencies. We do not, however, interpret these adjusted associations as evidence that personality scores can be used as direct selection or exclusion criteria. First, the residual variance in Honesty–Humility after adjusting for the Dark Triad is, in this analytic framework, a residualized observed-score quantity, not a measured latent fairness/sincerity disposition; second, sex-stratified analyses showed that the most informative trait predictors differ between men and women, particularly for gender-harassment perpetration tendencies; and third, harassment is shaped by structural and cultural factors that no individual-difference measure can substitute for. Within these caveats, broader preventive strategies—training programs that emphasize ethical decision-making, perspective-taking, and respect in hierarchical relationships, alongside organizational reforms that reduce power asymmetries and improve reporting systems—remain the principal policy levers indicated by this body of work. Personality assessment may complement, but should not replace, such systemic interventions. We explicitly note here that the author’s commercial activity in the personality-assessment industry (see Conflict of Interest) is independent of this study’s instruments (HEXACO-60 and SD3-J) and that the present findings are not used to validate, develop, or market any product.

### Limitations

Several limitations should be considered when interpreting the present findings. First, the study employed a cross-sectional design, which precludes causal inference. It cannot be determined whether personality traits drive subsequent harassment perpetration tendencies, whether harassment perpetration tendencies feed back into self-reported personality, or whether both reflect unmeasured third variables (e.g., organizational climate, leadership structure). Second, all variables were assessed by self-report at a single time point, raising the possibility of common method variance, social-desirability responding, and—particularly relevant here—underreporting of socially undesirable behaviors despite de-identification. Multi-method designs incorporating peer reports, supervisor evaluations, or behavioral indicators would strengthen future work. Third, the sample was recruited through the Lancers crowdsourcing platform and consisted of currently employed Japanese adults; representativeness across occupational sectors, hierarchical levels, employment types, and organizational sizes cannot be guaranteed. Industry, organizational size, and managerial status were not systematically assessed and may moderate personality–harassment associations. Fourth, the power-harassment items used in this study (Tou et al., 2017) presuppose a supervisory or otherwise hierarchical relational context with subordinates, but managerial status was not assessed at intake; it is therefore plausible that, for some respondents, the relational conditions presupposed by the items are not present. This sample–construct mismatch is a fundamental construct-validity concern that cannot be fully resolved through post-hoc adjustment within this dataset, and we acknowledge it explicitly: the power-harassment results should be read as applying to the population of currently employed Japanese adults responding to these items as written, not specifically to confirmed supervisors. Future replications should either restrict the sample to confirmed supervisors or include managerial status (subordinate count, reporting line, formal authority) as a covariate or stratifying variable. Fifth, the harassment outcomes in this study are composite indices rather than pure behavioral-frequency counts. The power-harassment composite, in particular, bundles behavioral enactment, attitudinal endorsement, and a supervisor-centered interpersonal-climate component (subordinate constriction and silence around the respondent) that conceptually reflect distinct constructs. Subscale-level analyses cannot be reproduced from the publicly archived file because that file retains only composite scores. Findings should accordingly be read as referring to perpetration-related composites rather than to behavioral perpetration in a narrow sense, and a focused replication that explicitly decomposes the composite by subscale is warranted. Sixth, the inferential framework used here is hierarchical multiple regression on observed (manifest) variables, and the H–H ‘incremental association’ should be read as such. The claim that residual H–H variance after adjustment for the Dark Triad indexes a substantively distinct moral-personality construct (rather than absorbing other content shared with H–H but not with Dark Triad) cannot be adjudicated within this analytic framework. A latent-variable approach—e.g., a bifactor or hierarchical CFA in which a general ‘antagonism’ factor and a specific H–H factor are jointly estimated—would be required to test that specificity claim. Seventh, sex-stratified estimates differ in precision (*n* = 133 men vs. 220 women) and are not derived from a formal cross-sex equality test; the divergence in male versus female predictor structure should accordingly be read as a hypothesis-generating pattern rather than as a confirmed moderation effect. Finally, although the study contributes data from a Japanese employee sample, it does not permit conclusions about cross-cultural universality. Direct comparative designs using harmonized measures and matched occupational samples are needed before claims of cultural specificity or universality can be made. Taken together, these limitations underscore the need for longitudinal, multi-method, latent-variable, and cross-cultural research—and for replications that include managerial status and decompose the harassment composites—to clarify the robustness and scope of the observed associations.

### Future directions

Building on the present findings and their limitations, several avenues for future research are warranted. First, longitudinal and multi-wave designs are essential for clarifying temporal ordering among personality traits and harassment perpetration tendencies. Tracking employees over time would allow researchers to examine whether stable personality characteristics prospectively relate to later harassment perpetration tendencies, or whether workplace experiences reciprocally shape self-perceptions of personality. Such designs would substantially strengthen causal inference beyond cross-sectional associations. Second, future research should incorporate multi-method assessment strategies. Combining self-reports with peer evaluations, supervisor ratings, organizational records, or experimentally derived behavioral measures would help reduce common method bias and clarify the behavioral validity of personality–harassment associations. In particular, examining whether HEXACO traits relate to externally evaluated misconduct would provide stronger evidence for practical relevance. Third, cross-cultural comparative research is necessary to determine whether the observed associations are culturally specific or more generalizable. Although the present study contributes evidence from a Japanese employee sample, parallel investigations using comparable instruments across cultural contexts would allow direct tests of invariance. Such work would clarify whether the role of Honesty–Humility and Dark Triad traits in harassment perpetration tendencies differs across societies characterized by varying norms regarding hierarchy, collectivism, and gender roles. Fourth, future studies may benefit from including additional personality constructs. Incorporating Sadism from the Dark Tetrad could help disentangle distinct forms of antagonistic motivation, while examining prosocial traits such as empathy or altruism may clarify protective mechanisms. Moreover, direct comparisons with alternative personality frameworks, including the Big Five, would allow a more comprehensive evaluation of the incremental contribution of Honesty–Humility. Finally, integrating personality variables with organizational-level factors—such as leadership style, ethical climate, reporting systems, and power structures—would provide a more complete multi-level account of workplace harassment. Harassment is unlikely to be reducible to individual traits alone; therefore, future research should model interactions between dispositional and contextual influences. Together, these directions would advance a more nuanced and empirically grounded understanding of how personality structure relates to workplace harassment across cultural and organizational settings.

## Conclusion

This study examined the associations among HEXACO traits, Dark Triad traits, and self-reported workplace harassment perpetration tendencies in a sample of currently employed Japanese adults, and tested whether the predictor structure differed by sex. Three findings stand out. First, in adjusted multivariate models, psychopathy retained a positive association with power-harassment perpetration tendencies and was a stronger adjusted predictor of power-harassment than of gender-harassment perpetration tendencies, particularly among men. Second, adding the six HEXACO factors yielded a non-trivial increase in explained variance over the Dark Triad and demographic covariates (ΔR^2^ = 0.032 for power, 0.096 for gender), with Honesty–Humility (and, for gender harassment, Openness) negatively associated with both outcomes after adjustment. Third, sex-stratified analyses revealed a meaningful divergence in predictor structure: for gender-harassment perpetration tendencies, Openness was the dominant negative predictor among men, while Honesty–Humility was the dominant negative predictor among women. Two interpretive cautions are emphasized. We frame the residual Honesty–Humility variance after adjustment for the Dark Triad as an adjusted association, not as evidence of a separable latent fairness/sincerity factor distinct from antagonism—an inference that would require a latent-variable framework not estimated here. We also frame the negative Machiavellianism coefficient in adjusted gender-harassment models as a feature of multivariate estimation under correlated antagonistic predictors (it is consistent with the small negative bivariate correlation, not a sign reversal under adjustment), not as evidence of substantive ‘protection.’ Within these constraints, the findings are broadly consistent with patterns reported in Western contexts but do not by themselves support claims of cultural universality. Future research that integrates HEXACO and the Dark Triad in longitudinal, multi-method, and cross-cultural designs—and that includes managerial status as a covariate, decomposes the harassment composites, and uses latent-variable modeling—would substantially strengthen the inferential basis on which personality-informed harassment-prevention strategies can be designed.

## Data Availability

Participants were recruited through the Lancers crowdsourcing platform (https://www.lancers.jp). Lancers user identifiers were collected at intake solely to disburse participant compensation, were stored separately from questionnaire responses, and were permanently deleted after payment. The dataset retained for analysis and made publicly available is therefore a de-identified dataset; it contains no direct identifiers (names, email addresses, IP addresses, exact dates of birth, employer information, or job titles) and no indirect identifiers from the recruitment platform (Lancers user IDs). To further reduce re-identification risk in the public release, the geographic-area variable was generalized from the original 11 Japanese regions into three categories (Kanto, Kinki, Other), and only HEXACO-60 subscale scores, SD3-J subscale scores, power-harassment and gender-harassment scale scores, age in 10-year decade categories, binary gender, and the generalized geographic-area category are retained. Each (age decade, gender, area category) combination corresponds to a real-world reference population numbering at least in the hundreds of thousands of Japanese residents, so re-identification of any individual record would require auxiliary information that is not contained in the released file. The de-identified dataset is available at: https://github.com/etoki/paper/blob/main/harassment/raw.csv and the analysis code at: https://github.com/etoki/paper/tree/main/harassment.

## Footnotes

1
https://www.lancers.jp

## References

[ref1] Anglim J. Sojo V. Ashford L. J. Newman A. Marty A. (2019). Predicting employee attitudes to workplace diversity from personality, values, and cognitive ability. J. Res. Pers. 83:103865. doi: 10.1016/j.jrp.2019.103865

[ref2] Ashforth B. (1994). Petty tyranny in organizations. Hum. Relat. 47, 755–778. doi: 10.1177/001872679404700701

[ref3] Ashton M. C. Lee K. (2007). Empirical, theoretical, and practical advantages of the HEXACO model of personality structure. Personal. Soc. Psychol. Rev. 11, 150–166. doi: 10.1177/1088868306294907, 18453460

[ref4] Ashton M. C. Lee K. (2009). The HEXACO-60: a short measure of the major dimensions of personality. J. Pers. Assess. 91, 340–345. doi: 10.1080/00223890902935878, 20017063

[ref5] Ashton M. C. Lee K. de Vries R. E. (2014). The HEXACO honesty-humility, agreeableness, and emotionality factors: a review of research and theory. Personal. Soc. Psychol. Rev. 18, 139–152. doi: 10.1177/1088868314523838, 24577101

[ref6] Ashton M. C. Lee K. Perugini M. Szarota P. de Vries R. E. Di Blas L. . (2004). A six-factor structure of personality-descriptive adjectives: solutions from Psycholexical studies in seven languages. J. Pers. Soc. Psychol. 86, 356–366. doi: 10.1037/0022-3514.86.2.356, 14769090

[ref7] Babiak P. Hare R. D. (2006). Snakes in Suits: When Psychopaths go to work. New York, NY: Regan Books/HarperCollins.

[ref8] Barnes C. M. Lucianetti L. Bhave D. P. Christian M. S. (2015). “You wouldn’t like me when I’m sleepy”: leaders’ sleep, daily abusive supervision, and work unit engagement. Acad. Manag. J. 58, 1419–1437. doi: 10.5465/amj.2013.1063

[ref9] Baughman H. M. Dearing S. Giammarco E. Vernon P. A. (2012). Relationships between bullying behaviours and the dark triad: a study with adults. Pers. Individ. Differ. 52, 571–575. doi: 10.1016/j.paid.2011.11.020

[ref10] Boddy C. R. (2011). Corporate psychopaths, bullying and unfair supervision in the workplace. J. Bus. Ethics 100, 367–379. doi: 10.1007/s10551-010-0689-5

[ref11] Breevaart K. de Vries R. E. (2017). Supervisor's HEXACO personality traits and subordinate perceptions of abusive supervision. Leadersh. Q. 28, 691–700. doi: 10.1016/j.leaqua.2017.02.001

[ref12] Breevaart K. de Vries R. E. (2021). Followers’ HEXACO personality traits and preference for charismatic, relationship-oriented, and task-oriented leadership. J. Bus. Psychol. 36, 253–265. doi: 10.1007/s10869-019-09671-6

[ref13] Bushman B. J. Baumeister R. F. (1998). Threatened egotism, narcissism, self-esteem, and direct and displaced aggression: does self-love or self-hate lead to violence? J. Pers. Soc. Psychol. 75, 219–229. doi: 10.1037/0022-3514.75.1.219, 9686460

[ref14] Bushman B. J. Bonacci A. M. van Dijk M. Baumeister R. F. (2003). Narcissism, sexual refusal, and aggression: testing a narcissistic reactance model of sexual coercion. J. Pers. Soc. Psychol. 84, 1027–1040. doi: 10.1037/0022-3514.84.5.1027, 12757146

[ref15] Christie R. Geis F. L. (1970). Studies in Machiavellianism. New York, NY: Academic Press.

[ref16] Cleckley H. (1941). The mask of Sanity: An Attempt to Clarify some Issues about the so-Called Psychopathic Personality. St. Louis, MO: C. V. Mosby.

[ref17] Cohen J. (1988). Statistical Power Analysis for the Behavioral Sciences. 2nd Edn Hillsdale, NJ: Routledge.

[ref18] Costa P. T. Terracciano A. McCrae R. R. (2001). Gender differences in personality traits across cultures: robust and surprising findings. J. Pers. Soc. Psychol. 81, 322–331. doi: 10.1037/0022-3514.81.2.322, 11519935

[ref19] Dåderman A. M. Ragnestål-Impola C. (2019). Workplace bullies, not their victims, score high on the dark triad and extraversion, and low on agreeableness and honesty-humility. Heliyon 5:e02609. doi: 10.1016/j.heliyon.2019.e02609, 31667422 PMC6812214

[ref20] Grijalva E. (2015). Narcissism and counterproductive work behavior (CWB): meta‐analysis and consideration of collectivist culture, big five personality, and narcissism's facet structure. Appl. Psychol. 64, 93–126. doi: 10.1111/apps.12025

[ref21] Hare R. D. (2003). Manual for the Hare Psychopathy Checklist–Revised. 2nd Edn Toronto, ON, Canada: Multi-Health Systems.

[ref22] Henrich J. Heine S. J. Norenzayan A. (2010). The weirdest people in the world? Behav. Brain Sci. 33, 61–83. doi: 10.1017/S0140525X0999152X, 20550733

[ref23] Howard M. C. Van Zandt E. C. (2020). The discriminant validity of honesty-humility: a meta-analysis of the HEXACO, big five, and dark triad. J. Res. Pers. 87:103982. doi: 10.1016/j.jrp.2020.103982

[ref24] Jonason P. K. Slomski S. Partyka J. (2012). The dark triad at work: how toxic employees get their way. Pers. Individ. Differ. 52, 449–453. doi: 10.1016/j.paid.2011.11.008

[ref25] Jones D. N. Paulhus D. L. (2009). “Machiavellianism,” in Handbook of Individual Differences in social Behavior, eds. Leary M. R. Hoyle R. H. (New York, NY: Guilford Press), 93–108.

[ref26] Kobayashi A. Tanaka K. (2010). Development of the gender harassment scale. Japan. J. Indust. Org. Psychol. 24, 15–27. doi: 10.32222/jaiop.24.1_15

[ref27] Koshinaka K. (2020). Do the dark triad personalities dislike others and avoid being disliked by others? Bull. Tokai Gakuen Univ., 30, 57–78. Available online at: http://repository.tokaigakuen-u.ac.jp/dspace/handle/11334/1903

[ref28] LeBreton J. M. Shiverdecker L. K. Grimaldi E. M. (2018). The dark triad and workplace behavior. Annu. Rev. Organ. Psych. Organ. Behav. 5, 387–414. doi: 10.1146/annurev-orgpsych-032117-104451

[ref29] Lee K. Ashton M. C. (2005). Psychopathy, Machiavellianism, and narcissism in the five-factor model and the HEXACO model of personality structure. Pers. Individ. Differ. 38, 1571–1582. doi: 10.1016/j.paid.2004.09.016

[ref30] Lee K. Ashton M. C. de Vries R. E. (2005). Predicting workplace delinquency and integrity with the HEXACO and five-factor models of personality structure. Hum. Perform. 18, 179–197. doi: 10.1207/s15327043hup1802_4

[ref31] Lee K. Ashton M. C. Wiltshire J. Bourdage J. S. Visser B. A. Gallucci A. (2013). Sex, power, and money: prediction from the dark triad and honesty-humility. Eur. J. Personal. 27, 169–184. doi: 10.1002/per.1860

[ref32] Lee Y. Berry C. M. Gonzalez-Mule E. (2019). The importance of being humble: a meta-analysis and incremental validity analysis of the relationship between honesty-humility and job performance. J. Appl. Psychol. 104, 1535–1546. doi: 10.1037/apl0000421, 31192647

[ref33] Lynam D. R. Derefinko K. J. (2006). “Psychopathy and personality,” in Handbook of Psychopathy, ed. Patrick C. J. (New York, NY: Guilford Press), 133–155.

[ref34] Masui K. Ura M. (2018). Adaptive strategies of “dark” individuals. Japan. Psychol. Rev. 61, 330–343. doi: 10.24602/sjpr.61.3_330

[ref35] Mathieu C. Hare R. D. Jones D. N. Babiak P. Neumann C. S. (2013). Factor structure of the B-scan 360: a measure of corporate psychopathy. Psychol. Assess. 25, 288–293. doi: 10.1037/a0029262, 22775409

[ref36] Morales N. A. (2020). Gender and the HEXACO model of Personality: Meta-Analysis, Measurement Equivalence, and Specification of Agency as a core construct of Investment (Master’s thesis). University of Illinois at Urbana-Champaign. Available online at: http://hdl.handle.net/2142/108140

[ref37] Morf C. C. Rhodewalt F. (2001). Unraveling the paradoxes of narcissism: a dynamic self-regulatory processing model. Psychol. Inq. 12, 177–196. doi: 10.1207/S15327965PLI1204_1

[ref38] Moshagen M. Hilbig B. E. Zettler I. (2018). The dark core of personality. Psychol. Rev. 125, 656–688. doi: 10.1037/rev0000111 29999338

[ref39] O'Boyle E. H. Forsyth D. R. Banks G. C. McDaniel M. A. (2012). A meta-analysis of the dark triad and work behavior: a social exchange perspective. J. Appl. Psychol. 97, 557–579. doi: 10.1037/a0025679, 22023075

[ref40] Oda R. Matsumoto-Oda A. (2022). HEXACO, dark triad and altruism in daily life. Personal. Individ. Differ. 185:111303. doi: 10.1016/j.paid.2021.111303

[ref41] Paulhus D. L. Williams K. M. (2002). The dark triad of personality: narcissism, Machiavellianism and psychopathy. J. Res. Pers. 36, 556–563. doi: 10.1016/S0092-6566(02)00505-6

[ref42] Pletzer J. L. Bentvelzen M. Oostrom J. K. de Vries R. E. (2019a). Comparing domain- and facet-level relations of the HEXACO personality model with workplace deviance: a meta-analysis. Personal. Individ. Differ. 152:109539. doi: 10.1016/j.paid.2019.109539, 38826717

[ref43] Pletzer J. L. Bentvelzen M. Oostrom J. K. de Vries R. E. (2019b). A meta-analysis of the relations between personality and workplace deviance: big five versus HEXACO. J. Vocat. Behav. 112, 369–383. doi: 10.1016/j.jvb.2019.04.004

[ref44] Pletzer J. L. Oostrom J. K. de Vries R. E. (2021). HEXACO personality and organizational citizenship behavior: a domain- and facet-level meta-analysis. Hum. Perform. 34, 126–147. doi: 10.1080/08959285.2021.1891072

[ref45] Raskin R. Terry H. (1988). A principal-components analysis of the narcissistic personality inventory and further evidence of its construct validity. J. Pers. Soc. Psychol. 54, 890–902. doi: 10.1037/0022-3514.54.5.890, 3379585

[ref46] Schmitt D. P. Realo A. Voracek M. Allik J. (2008). Why can’t a man be more like a woman? Sex differences in big five personality traits across 55 cultures. J. Pers. Soc. Psychol. 94, 168–182. doi: 10.1037/0022-3514.94.1.168 18179326

[ref47] Shimotsukasa T. Oshio A. (2017). Development and validation of the Japanese version of the short dark triad (SD3-J). Japan. J. Personal. 26, 12–22. doi: 10.2132/personality.26.1.2

[ref48] Shimotsukasa T. Oshio A. (2019). The relationship between the dark triad and interpersonal manipulation strategies. Japan. J. Personal. Adv. 28, 119–127. doi: 10.2132/personality.28.2.3

[ref49] Takada T. (2023). The relationship between gambling disorder tendencies and the dark triad in Japanese gamblers. Japan. J. Personal. 32, 82–84. doi: 10.2132/personality.32.2.6

[ref50] Tamura A. Oshio A. Tanaka K. Masui K. Jonason P. K. (2015). Development, reliability, and validity of the Japanese version of the dark triad dirty dozen (DTDD-J). Jpn. J. Pers. 24, 26–37. doi: 10.2132/personality.24.26

[ref51] Thielmann I. Moshagen M. Hilbig B. E. Zettler I. (2021). On the comparability of basic personality models: meta-analytic correspondence, scope, and orthogonality of the big five and HEXACO dimensions. Eur. J. Personal. 36, 318–348. doi: 10.1177/08902070211026793

[ref52] Tomii M. Oshio A. (2024). The relationship between the dark tetrad and loneliness: the mediating role of exclusion experiences. Japan. J. Personal. 33, 65–74. doi: 10.2132/personality.33.1.9

[ref53] Tou K. Tsuda A. Nii M. Yamahiro T. Irie M. (2017). “Development of new power harassment questionnaire in workplace,” in Proceedings of the 81st Annual Convention of the Japanese Psychological Association, vol. 81 (), 17.

[ref54] Tsuno K. Kawakami N. Tsutsumi A. Shimazu A. Inoue A. Odagiri Y. . (2015). Socioeconomic determinants of bullying in the workplace: a national representative sample in Japan. PLoS One 10:e0119435. doi: 10.1371/journal.pone.0119435, 25751252 PMC4353706

[ref55] Vize C. E. Lynam D. R. Collison K. L. Miller J. D. (2018). Differences among dark triad components: a meta-analytic investigation. Pers. Disord. 9, 101–111. doi: 10.1037/per0000222, 27736106

[ref56] Wakabayashi A. (2014). A sixth personality domain that is independent of the big five domains: the psychometric properties of the HEXACO personality inventory in a Japanese sample. Jpn. Psychol. Res. 56, 211–223. doi: 10.1111/jpr.12045

[ref57] Zeigler-Hill V. Besser A. Morag J. Campbell W. K. (2016). The dark triad and sexual harassment proclivity. Pers. Individ. Differ. 89, 47–54. doi: 10.1016/j.paid.2015.09.048

[ref58] Zettler I. Thielmann I. Hilbig B. E. Moshagen M. (2020). The nomological net of the HEXACO model of personality: a large-scale meta-analytic investigation. Perspect. Psychol. Sci. 15, 723–760. doi: 10.1177/1745691619895036, 32324493

